# Round the Hospitals

**Published:** 1921-06-25

**Authors:** 


### ROUND THE HOSPITALS.

The foundation-stone of the new Florence Nightingale Training School for Nurses, which is dedicated to the memory of 284 American nurses who gave their lives during the war, was laid on June 5 at Bagatelle in the Bordeaux suburb of Talence. The money for this building has been subscribed by American nurses. It is designed to carry on and largely develop a work founded twenty years ago by Dr. Anna Hamilton to raise the standard of nurse-training in France. The stone was laid by Miss Helen Scott Hay, of Savanna, Illinois, Chief Nurse of the American Red Cross in Europe, on behalf of Miss Clara Noyes, chairman of the Fund for the Memorial. An influential gathering of Frenchmen and Americans was present, and Admiral Magruder, American Naval Attaché in France, was in charge of the ceremonies.

The number of policies issued last year by the Royal National Pension Fund for Nurses was 777, while the number of policies surrendered was over a thousand, held by 911 nurses. The temptation to withdraw savings at any moment of difficulty has always proved hard to resist, though it often happens that the withdrawal is followed by the purchase of a new policy. Annual premiums paid in amounted last year to £891,184. On December 31, 1920, the Fund had 2,891 annuitants on its books, receiving at the rate of £76,000 a year, the average annuity being slightly over £26 10s. The invested funds have now reached a total of over two millions. The operations of the Junius S. Morgan Benevolent Fund have been fruitful for good among the members. Many grants were given to augment the pensions of the older and invalid members. Many grants have been given to assist nurses with their premiums in illness and convalescence, and assistance has also been given in emergencies of many kinds. The punctual payment of the shilling contribution towards this Fund which every policyholder is asked to give would greatly facilitate this good work. But should not the contribution now be two shillings?

The most interesting announcement of the afternoon on the occasion of the Prize Distribution at St. Thomas's Hospital on June 21 was that of the gift to the President the Duke of Connaught of a cheque for £2,120 which has been collected by the matron, the sisters, nurses, and probationers from amongst their friends, in love of their hospital and with sympathy in the time of anxiety for its welfare. A pleasant feature of the gift is that none of the money has been contributed by any of the nursing staff; this was a condition distinctly laid down, though the nurses delightedly evaded it by performing personal services in return for a contribution, such as hat-trimming, sweet-making, fancy work, etc. The sum raised is remarkable, and this success led the treasurer, Sir Arthur Stanley, characteristically to remark that in view of what sisters and nurses have thus shown they can do, it seems high time that the management of the hospital was handed over to the ladies. Justly, Nightingale nurses came in for a good deal of attention in the speeches of the afternoon. Sir Arthur recalled that Queen Victoria, in opening the present hospital buildings fifty years ago, expressed the hope that there would be an adequate supply of careful and well-trained nurses. Sir Cuthbert Wallace emphasised the recognition by the Nightingale School that there were two sides to every scientific education, the academic and the technical. The Nightingale School was one of the first to develop the former, as it was in fact the first to frame a system of nurse-training. The Nightingale nurse has nobly upheld the traditions of her school and her hospital, and there is no doubt that she will continue to reach even the high standard set before her.

---

It is now officially announced that, consequent on Miss Vera Matheson's resignation for private reasons of the office of Secretary to the Irish Board of the College of Nursing, Miss M. F. Chisholm has been elected to the vacancy, and that her appointment will date from September 1, 1921. Miss Chisholm, who has had considerable nursing and administrative experience in Ireland, was trained at the Middlesex Hospital and the Mater Infirmorum Hospital, Belfast. She became matron of a private cottage hospital at Inverness-shire, and for six years was Queen's nurse in Ireland. She was then appointed Inspector of the Queen Victoria Jubilee Institute in Ireland, which post she has held for eleven years, during three of which she was acting superintendent of the Irish branch.

---

In response to the letter sent out by the General Nursing Council of England and Wales to all training-schools for nurses, with a copy of the new Nursing Syllabus, the chairman of the Bedford County Hospital replies that the Board of Management wishes the hospital to be placed on the List of Approved Institutions for the Training of Nurses, and is therefore prepared to adopt the curriculum of training laid down in the Syllabus. The chairman adds, however, that the Board desires to express an emphatic opinion that "the draft Syllabus is of so technical and advanced a character as to make its application difficult, if not impracticable, in the case of the majority of provincial hospitals." We believe the Board will come to recognise that the difficulties in working up to the Syllabus are more apparent than real.

The Royal Bucks Hospital celebrated with great *éclat* the completion by the matron, Miss M. G. Vaughan, of twenty-five years' service. The Marquis of Lincolnshire, Lord Lieutenant of Bucks, in the presence of a large company, presented Miss Vaughan with an illuminated address, a purse and cheque for £220, with an album containing the names of the subscribers. The inscription records the "continuous, loyal and valuable service" of Miss Vaughan, four years as sister and twenty-one years as matron. One of the 700 contributors, an old-age pensioner "Rebecca," sent two stamps and expressed her gratitude for the attention she had received in the hospital. Dr. Perrin expressed on behalf of the medical staff the hope that matron would celebrate her jubilee in the hospital. During the war the hospital had no house surgeon. Miss Vaughan stepped into the breach and was practically in all but name house surgeon as well as matron. The Lord Lieutenant spoke of her as a true English woman. He offered the testimonial as a token of thanks from the county for her great and good services for so many years. The nursing staff presented a silver bowl and the domestic staff a pair of silver candlesticks.

---

The colour bar has prohibited, we regret to learn, the South African Trained Nurses Association from admitting to its ranks a coloured lady who is fully trained and holds a certificate qualifying her for membership. There does not appear to be any rule limiting membership to fully trained nurses of white European descent, but as application for admission has been formally made by at least one coloured applicant it was unanimously resolved at the last meeting to amend the constitution and definitely exclude coloured and native nurses. Overcrowding in nurses' homes appears to be at least as prevalent in South Africa as at home, and we note a recommendation of the S.A.T.N.A.: "That the practice of allowing day and night nurses to occupy the same bedrooms should not be allowed." Of all the forms of overcrowding this is perhaps the worst.

---

An important prosecution under the Midwives Act is reported from Portsmouth, where on May 24 Emily Reynolds was summoned at the instance of the Maternity and Child-Welfare Committee with habitually and for gain attending women in childbirth as midwife, not being certified under the Midwives Act. Mrs. Reynolds, who described herself on the doorplate as a certified maternity and surgical nurse, ran a nursing home in the Sultan Road, Landport, and three witnesses deposed that she led them to believe she was certified. A certified midwife, sixty-nine years of age and nearly blind, was called in either just before some of the births, at the births, or after. The witnesses gave evidence that they had been disgracefully neglected. On her undertaking to give up entirely any connection with maternity the defendant was let off with a fine of £5.

